# Real-Time Deep-Sea Mooring System with Inductive Telemetry and Multi-Sensor Integration: Deployment and Performance in the South China Sea

**DOI:** 10.3390/s26030937

**Published:** 2026-02-01

**Authors:** Tongmu Liu, Baocheng Zhou, Xinwen Zhang, Jianqing Peng, Hua Huang, Tianhao Jian

**Affiliations:** 1 South China Sea Marine Survey Center, Ministry of Natural Resources, Guangzhou 510300, China; liutongmu@126.com (T.L.); xinwenzh@foxmail.com (X.Z.); hhaostos@126.com (H.H.); 15737682122@163.com (T.J.); 2 Key Laboratory of Marine Environmental Survey Technology and Application, Ministry of Natural Resources, Guangzhou 510300, China; 3 Southern Marine Science and Engineering Guangdong Laboratory (Zhuhai), Zhuhai 511458, China; 4 School of Intelligent Systems Engineering, Shenzhen Campus of Sun Yat-Sen University, Shenzhen 518107, China; pengjq7@mail.sysu.edu.cn; 5 Guangdong Provincial Key Laboratory of Fire Science and Technology, Guangzhou 510641, China

**Keywords:** real-time mooring, inductive telemetry, South China Sea, ADCP, CTD, Tiantong satellite

## Abstract

Obtaining real-time data from the deep ocean remains a major challenge in marine observation. This study presents a deep-sea mooring system that integrates inductive telemetry (EUM6000 modules) with Tiantong satellite communication to achieve real-time, long-term hydrological monitoring in the South China Sea. The system incorporated 25 sensors, including CTDs and ADCPs, and was deployed at a depth of 1247 m. Over one year of continuous operation, it maintained a data reception rate > 90%, with a latency of <15 min from seabed to shore. Compared to acoustic-based systems, the inductive telemetry design significantly improved energy efficiency and reliability. The high-resolution multi-sensor data provide valuable insights into ocean dynamics and support applications in climate research and disaster early warning. This system offers a robust solution for real-time deep-sea observation and serves as a reference for future ocean network development.

## 1. Introduction

The ocean is a vital component of the Earth system, harboring abundant resources and playing a crucial role in regulating the global climate. The deep ocean, constituting the bulk of the ocean, has long been at the forefront of research in physical oceanography, marine geology, and global climate change studies, with its internal hydrodynamic processes, material and energy exchanges, and ecological evolution serving as key focal points [1]. Acquiring long-term, continuous, high-resolution in situ observation data from the deep ocean holds irreplaceable scientific value for profoundly understanding ocean circulation structures, mesoscale eddy dynamics, internal ocean mixing processes, and accurately predicting the evolution trends of climate models [2,3,4].

Ocean observation plays a crucial role in understanding ocean processes, climate change prediction, environmental monitoring, and resource management. Long-term, continuous ocean data collection is vital for studying physical oceanography, biogeochemical cycles, and ecosystem dynamics. Traditionally, ocean observation relies on mooring systems, including surface moorings [5] and subsurface mooring systems [6]. Surface mooring systems enable real-time data transmission via buoys and satellite communications but are susceptible to surface weather disturbances, waves, and human interference. Subsurface mooring systems, typically deployed in deep waters, avoid surface disruptions but can only retrieve data upon system recovery, lacking real-time capability. This limitation restricts their application in operational oceanography and real-time early warning systems [7,8].

To achieve real-time acquisition of deep-sea data, research institutions worldwide have developed multiple technical approaches, with the core challenge being the transmission of underwater data to shore-based centers [9,10]. Morrison et al. [11] developed the McLane Moored Profiler (MMP), which operates by utilizing a towing drive system to autonomously ascend and descend along an anchor mooring while simultaneously conducting measurements and observations. Send et al. [12] developed the SeaCycler autonomous ocean profiling system. This system employs an underwater winch to deploy and retrieve cables, controlling the ascent and descent of communication buoys. The buoys are raised to the surface only during data transmission. Similar approaches were proposed by Xu [13], Lin [14], Yu [15], and others [16,17]. Tian et al. [18] designed a low-cost, reusable underwater mooring system capable of periodic data uploads, primarily employing inductive coupling and timed release of communication buoys.

Zhang [19] developed a timed communication mooring that releases data-carrying communication buoys at predetermined intervals. After surfacing, the buoys transmit data via satellite. Wang et al. [20] developed a real-time communication mooring integrating acoustic and satellite communication. Its operating principle involves using acoustic communication technology to upload deep-sea data to surface buoys, which then relay the data back via satellite systems such as Iridium or BeiDou. Despite significant progress in real-time communication mooring technology, challenges remain in areas such as deep-water deployment, multi-sensor integration, and satellite communication capabilities.

With the rapid advancement of satellite communications, underwater sensing, and data transmission technologies, establishing real-time deep-sea observation systems has become a key development direction in international ocean observation [21]. Among these, inductive coupling transmission technology utilizes seawater as a medium and mooring cables as conductors to achieve non-contact penetration for electrical power and data transmission [22]. This provides a critical technical pathway for enabling long-term, reliable, high-speed data transfer between underwater observation platforms and surface floats [23]. Concurrently, the establishment of China’s satellite mobile communication systems [24], such as Tiantong-1, provides stable and reliable long-distance communication support for transmitting deep-sea observation data back to shore-based receiving centers from remote locations [5]. Integrating inductive coupling underwater transmission with Tiantong satellite remote communication to establish an end-to-end real-time data link spanning “seabed-surface-shore stations” represents a highly promising innovative solution for overcoming the bottleneck of real-time deep-sea observation data transmission.

This paper presents the design, deployment, and validation of a deep-sea real-time transmission mooring system based on inductive coupling transmission technology and Tiantong satellite communication. This paper will detail the overall design, core technology integration plan, and implementation approaches for key technologies of this real-time communication hydrographic mooring system. It will focus on analyzing field test data from the South China Sea to validate the system’s long-term stability, data reliability, and overall performance in real ocean environments. This aims to provide critical technical support and engineering practice references for constructing next-generation deep-sea real-time observation networks.

## 2. System Design and Methodology

### 2.1. Overall System Design

The deep-sea hydrographic mooring system primarily consists of a communication buoy, main buoy body, inductively coupled cable, acoustic release mechanism, and composite anchor. Components are interconnected via inductively coupled steel cables, stainless steel anchor chains, Kevlar mooring ropes, inductively coupled anti-twist modules, shackles, and other connectors. The communication buoy is equipped with a Tiantong satellite real-time communication system.

Two 75K ADCPs are mounted on the main buoy, transmitting data via watertight cables to the inductive telemetry module compartment installed on the main buoy. Inductively coupled steel cables are attached above and below the main float, carrying inductively coupled sensors such as temperature-depth, temperature-salinity-depth, and current meters. Data collected by these sensors at various water layers is transmitted via inductively coupled steel cables and the inductively coupled data transmission system to the inductive telemetry module compartment on the main float. The inductive telemetry module compartment then transmits the integrated and packaged datasets in batches via inductively coupled cables to the data acquisition and control module of the satellite communication buoy.

**Figure 1:**
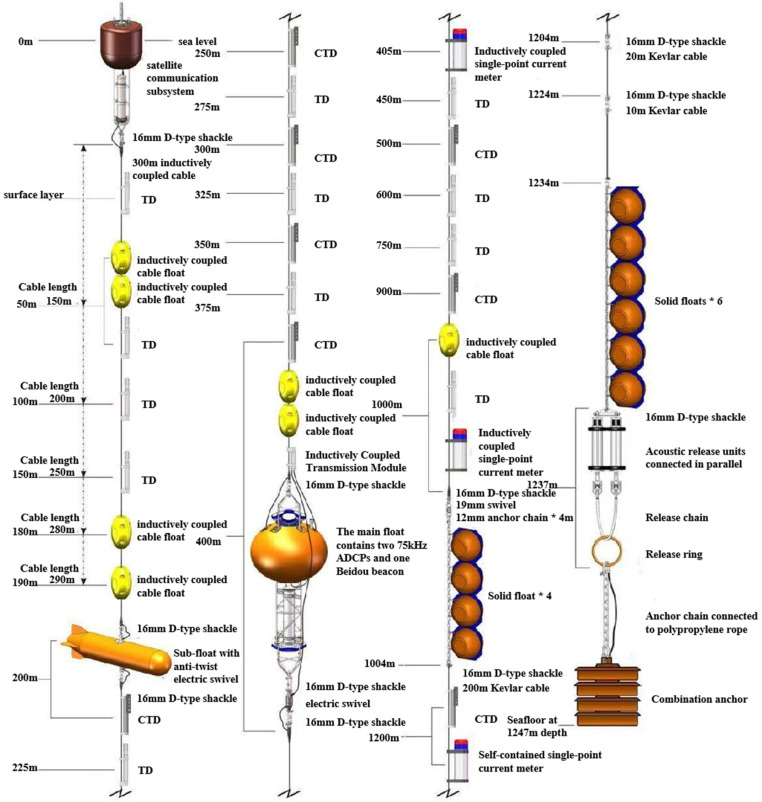
Overall design of coupled transmission mooring system.

The mooring system anchorage is designed as a three-tier structure with a communication buoy, sub-float, and main float. The system structure diagram is shown in Figure 1.

### 2.2. Sensor Integration and Configuration

The mooring system was designed to capture high-resolution, multi-parameter hydrological data throughout the entire water column. To achieve this, a suite of 25 sensors was strategically integrated onto the inductive coupling telemetry cable, forming a distributed observation array. The selection of sensors prioritized long-term stability, high accuracy, and seamless compatibility with the inductive data transmission system. The specific types, models, quantities, and key specifications of the deployed sensors are summarized in Table 1.

#### 2.2.1. Stratified Hydrological Parameters

The core temperature and salinity data were provided by nineteen instruments: seven SBE37-SM MicroCAT CTD sensors (Sea-Bird Scientific, Bellevue, WA, USA) and twelve OceanPlot TD-IM inductive temperature-depth sensors (OceanPlot, Hangzhou, China). The SBE37-SM sensors were chosen for their proven high accuracy and long-term stability in measuring the fundamental parameters of conductivity, temperature, and pressure, from which salinity is derived. The TD-IM sensors provided complementary high-precision temperature and depth measurements, significantly increasing the spatial density of the thermal structure observation.

These sensors were distributed at critical depths along the mooring cable, with a higher concentration within the upper ocean (0–400 m) to resolve the strong vertical gradients of the thermocline and halocline. This configuration allows for the detailed reconstruction of the water column’s thermodynamic structure and the identification of fine-scale processes such as internal waves and intrusions.

#### 2.2.2. Current Measurement Strategy

A dual-method approach was employed to measure ocean currents:

Point Measurements: Two ZPulseIM inductive single-point current meters (Aanderaa, Bergen, Norway) were installed at predetermined depths (405 m and 1000 m) to provide long-term, stable records of current velocity and direction at specific, strategic locations.

Profile Measurements: Two 75 kHz Teledyne Workhorse Long Ranger ADCPs (Teledyne RD Instruments, Poway, CA, USA) were mounted on the main subsurface buoy at approximately 400 m depth. This configuration was designed to maximize the coverage of the water column: one ADCP was oriented upwards to profile currents from the buoy towards the surface, and the other was oriented downwards to measure currents from the buoy towards the seabed. This setup provides a comprehensive view of the current shear and structure over most of the water column, which is essential for understanding dynamics like eddies and deep currents.

#### 2.2.3. Data Integration via Inductive Telemetry

A key innovation of this system is the seamless integration of these diverse sensors via the EUM6000 inductive telemetry modules. Each sensor equipped with an IM interface communicates through the single, specially designed inductive coupling cable. This design eliminates the need for individual data cables for each sensor, drastically reducing the system’s complexity, weight, and potential points of failure. The EUM6000 modules aggregate data from all sensors, packetize them, and relay the integrated datasets upwards to the surface communication buoy for satellite transmission. This integrated architecture ensures the synchronous acquisition and real-time transmission of all hydrological parameters, making the entire water column remotely accessible.

### 2.3. Inductive Telemetry Module

#### 2.3.1. Working Principle of Inductive Coupling

The inductive telemetry technology serves as the critical data link between submerged sensors and the data acquisition unit in this mooring system. Its operation is based on the fundamental principle of electromagnetic induction, enabling robust and efficient data transmission through a standard, plastic-coated steel mooring cable without the need for physical electrical contacts [25].

**Figure 2:**
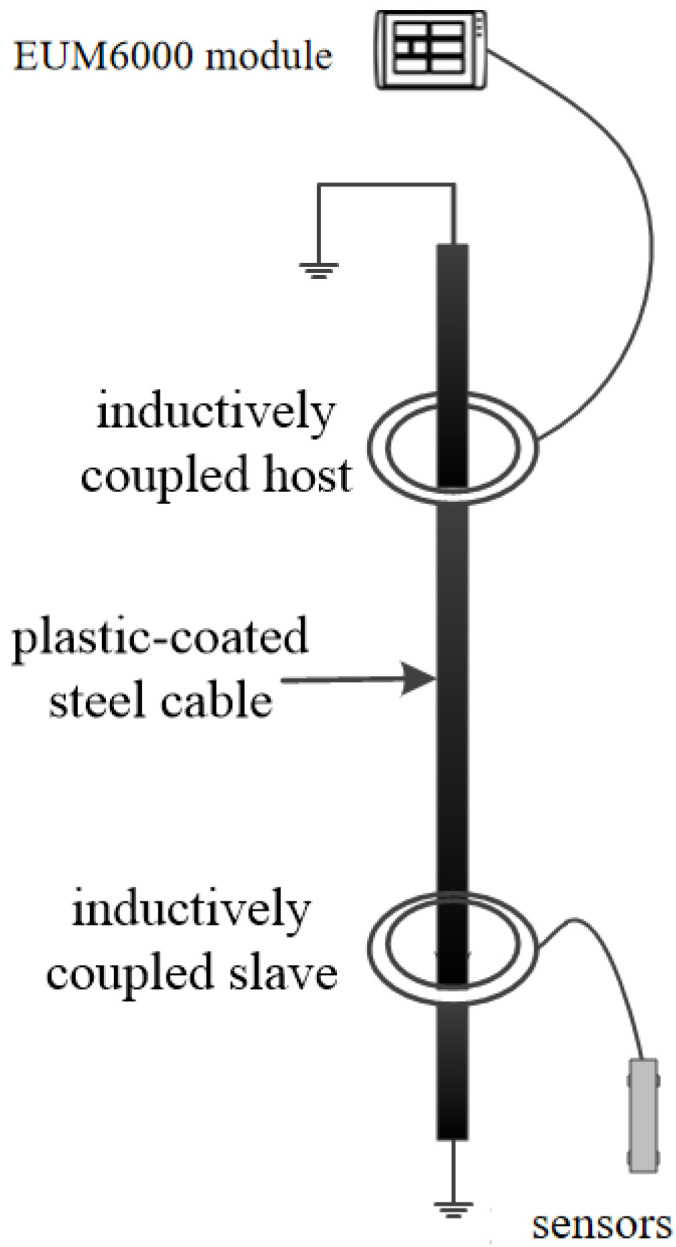
Schematic diagram of inductive coupling transmission.

As illustrated in Figure 2, the system primarily consists of two key components: an Inductively Coupled Master (Host) unit, typically integrated within the data logger, and multiple Inductively Coupled Slave units connected to individual sensors (e.g., pressure sensors) [6]. Each unit contains a ferrite core around which a coil is wound, forming a magnetic coupling circuit.

The working principle can be described in three steps:

(1) Signal Modulation and Magnetic Field Generation: At the transmitting end (e.g., the slave unit connected to a pressure sensor), the digital data signal is first modulated. This modulated electrical signal is then fed into the coupling coil, generating an alternating magnetic field around the coil according to Ampère’s circuital law. This magnetic field is confined and guided by the high magnetic permeability ferrite core.
(2) Magnetic Flux Coupling via the Cable: The key innovation lies in using the steel mooring cable itself as a conduit for the magnetic flux. The alternating magnetic field produced by the transmitting coil magnetically couples with the steel cable. The cable, acting as a low-reluctance path, channels this magnetic flux over long distances with minimal loss. This process effectively transforms the entire cable into a “virtual wire” for magnetic signals.
(3) Signal Reception and Demodulation: At the receiving end (e.g., the master unit), the alternating magnetic flux flowing through the cable induces an electrical current in the receiving coil, following Faraday’s law of induction. This induced electrical signal is then demodulated to recover the original digital data transmitted by the sensor.

This non-contact, magnetic induction-based method offers significant advantages over other underwater communication techniques:

High Reliability: Unlike acoustic telemetry, which is susceptible to noise, multipath interference, and absorption losses in water, inductive coupling provides a stable and predictable channel because magnetic fields are less affected by the aquatic environment.

Low Power Consumption: The power required for short-range inductive coupling is substantially lower than that for acoustic modems, which is crucial for long-term deployments.

Simplified Integration: A single cable serves both the mechanical mooring function and the data/power transmission bus, drastically reducing system complexity compared to using multiple dedicated electrical cables for each sensor.

In this system, the EUM6000 modules implement this principle, ensuring reliable, real-time transmission of data from all distributed sensors to the central data logger for subsequent processing and satellite transmission.

#### 2.3.2. EUM6000 Module Specifications and Performance

The EUM6000 inductive telemetry module is the core component that implements the working principle described above, serving as the data gateway for the entire sensor array. Its specifications are meticulously engineered to meet the demands of long-term, deep-sea observation, and its performance is a critical determinant of the system’s overall reliability. The key specifications of the EUM6000 module are summarized in Table 2.

The performance of the EUM6000 module was rigorously validated during the system’s development and sea trials, demonstrating several key advantages:

(1) High Data Fidelity: The module employs robust error-checking protocols to ensure data integrity. The use of electromagnetic induction avoids the high bit error rates typical of underwater acoustic channels, which was a primary factor in achieving the overall system data reception rate of >90% during the South China Sea deployment.
(2) Low-Latency Communication: Data transmission through the EUM6000 system is nearly instantaneous, with latency determined primarily by the signal propagation speed (approaching the speed of light in the cable medium). This is in stark contrast to acoustic telemetry, which suffers from slow propagation speeds (~1500 m/s), resulting in significant delays over long distances. This low latency is crucial for creating a synchronized, real-time profile of the water column.
(3) System Simplicity and Reliability: A significant performance metric is the mean time between failures (MTBF). The solid-state design of the EUM6000, with no moving parts and minimal underwater connectors, contributes to a high MTBF. This design eliminates the primary points of failure associated with traditional multi-core watertight cables, which are prone to leakage and damage under deep-sea pressure and dynamic loading.

In conclusion, the EUM6000 module is not merely a communication component but an enabler of the system’s core innovation. Its specifications for depth, data rate, and low power consumption, combined with its demonstrated performance in reliability and latency, directly support the successful integration of multiple sensors and the establishment of a robust real-time data link from the deep sea to the shore station.

### 2.4. Tiantong Satellite Communication System

The Tiantong-1 satellite system is a cornerstone of China’s independent space-based infrastructure, operating in geosynchronous Earth orbit (GEO) to provide secure mobile communication services over its coverage area, which encompasses the South China Sea. For deep-sea mooring systems requiring real-time data telemetry, the selection of a communication system must balance data rate, reliability, power consumption, and national security considerations. While systems like Iridium and Inmarsat offer global coverage, the Tiantong system was selected for this study due to its superior data rate compared to the BeiDou Short Message Service (SMS), lower operational cost, and critically, its status as a sovereign, secure communication asset.

#### 2.4.1. System Architecture and Link Budget

The communication chain follows a standardized architecture: Mooring System → Tiantong User Terminal (on surface buoy) → Tiantong GEO Satellite → Ground Gateway Station → Shore-based Data Center. The user link operates in the S-band, which offers a favorable balance between antenna size, atmospheric attenuation, and beam width, making it suitable for small marine platforms. The terminal employed in this system features a directional antenna with a servo-stabilized gimbal mechanism. This mechanism is imperative for maintaining a high-quality link under dynamic sea states. An integrated Inertial Measurement Unit (IMU) provides real-time attitude data (pitch, roll) to an Application Processor (AP), which calculates compensation angles to ensure precise antenna pointing towards the satellite, achieving a tracking accuracy of better than 2° even with buoy tilts of ±30°.

#### 2.4.2. Communication Protocol Stack and Data Handling

The system implements a structured protocol stack to manage the complexity of satellite communication. Interaction with the Tiantong modem is governed by a set of AT commands sent via a UART interface. Key commands used in our implementation include AT + CSQ (to query signal strength),AT + CGREG = 1 (to register with the packet-switched network), and ATD*98*4# (to initiate a PPP dial-up connection) [5].

**Figure 3:**
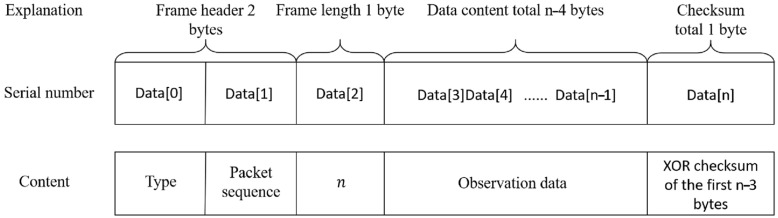
Encapsulated data frame structure.

To efficiently utilize the available bandwidth and ensure data integrity, a custom application-layer protocol was designed, as illustrated in Figure 3.

Framing and Encapsulation: Large datasets, particularly from the ADCP, exceed the typical maximum transmission unit (MTU) of a single satellite packet. Therefore, data is fragmented into frames. Each frame consists of a Header, Payload, and Trailer.

Header: Contains a Start Delimiter, a Data Type identifier and a Frame Sequence Number.

Payload: Contains the actual sensor data.

Trailer: Contains a Cyclic Redundancy Check (CRC) or an XOR checksum computed over the entire frame to detect transmission errors.

Quality of Service (QoS) and Retransmission Mechanism: A critical aspect of the protocol is its QoS strategy to handle packet loss. The shore station monitors the sequence numbers of incoming frames. Upon detecting a gap, it issues a NACK (Negative Acknowledgement) command via the Tiantong return link, specifying the missing frame number. The buoy-side controller stores recent data packets in a buffer and retransmits the requested frame. This process is repeated for a predefined maximum number of attempts to ensure robustness without depleting power resources.

#### 2.4.3. Performance Metrics

In this deployment, the Tiantong terminal operated with an effective data rate of 9.6 kbps. The average latency for a data packet traveling from the buoy to the shore station was measured to be less than 15 min, which includes processing, satellite hop, and terrestrial network routing delays. The system’s link budget, designed with a sufficient margin, coupled with the automatic beam-tracking, resulted in a measured data delivery success rate of >90% during the sea trial, validating the effectiveness of the chosen communication architecture and protocols for real-time ocean observation.

### 2.5. Power System and Energy Consumption Analysis

To ensure the system can achieve long-term, continuous observation exceeding one year, we conducted a detailed analysis and design of the power requirements for the entire observation chain. The system adopts a distributed power supply strategy: the inductively coupled transmission link and satellite communication module are powered by independent battery packs, while the sampling power consumption of certain sensors (such as CTDs) is handled by their built-in batteries. This approach optimizes system redundancy and reliability.

Power consumption analysis for major components is as follows: The 12 TD-IM temperature-salinity-depth loggers and 7 CTD-IM inductively coupled modules, due to their low power characteristics, are projected to operate for over 28,000 h each. The two inductively coupled single-point current meters and the inductively coupled transmission module for the ADCP, operating on a 30 min cycle, are projected to last 60,000 h and 35,000 h, respectively.

The satellite communication module represents the core power consumption component of the entire system. During its one-hour operational cycle, the total power consumption for completing one inductively coupled data acquisition and satellite data transmission cycle is approximately 18 mAh. Based on this calculation, the equipped 238 Ah high-capacity battery can support continuous operation of this module for over 10,000 h (approximately 416 days), fully meeting the design life requirements. Considering the combined power consumption of all equipment and the distributed power supply solution, the entire observation system possesses the energy assurance to operate continuously and stably for over one year while executing its designated observation tasks.

## 3. Field Deployment and Validation

### 3.1. Pre-Deployment Preparation and Laboratory Testing

Prior to the sea deployment, all sensors, including the CTDs and TD sensors, were calibrated against a shipboard SBE 911 + CTD system. The results showed a root mean square deviation (RMSD) of 0.003 PSU for salinity and 0.002 °C for temperature, ensuring data accuracy. The entire mooring system was assembled and tested dockside to verify the integrity of mechanical connections, the functionality of the acoustic release mechanism, and the end-to-end data flow from seabed sensors to the satellite transmission. A 72 h continuous test confirmed the stability of the inductive telemetry link and the power management system, with the measured average power consumption projecting a battery life exceeding 400 days.

### 3.2. Sea Trial Deployment: Procedure and Site Description

**Figure 4:**
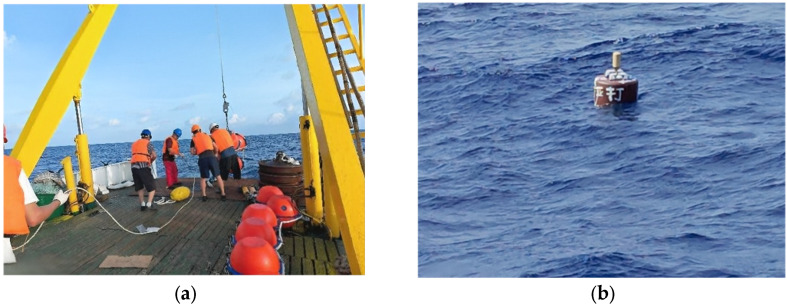
Deployment Site Diagram for Submerged Buoys. (**a**) Field Operation Diagram; (**b**) Tiantong Communications Buoy.

The mooring system was deployed on 22 June 2024. The deployment site was selected at a depth of 1247 m in the northern South China Sea, a region known for its strong internal solitary wave activities, providing an ideal environment for validating the system’s capability to capture dynamic processes. The deployment procedure followed the standard sequence: first, the surface communication buoy was lowered, followed by the controlled deployment of the mooring line with sensors, the main float, release and finally, the anchor weight. The field operation diagram for deploying the moored buoy is shown in Figure 4.

### 3.3. Data Reception and System Operational Status

**Figure 5:**
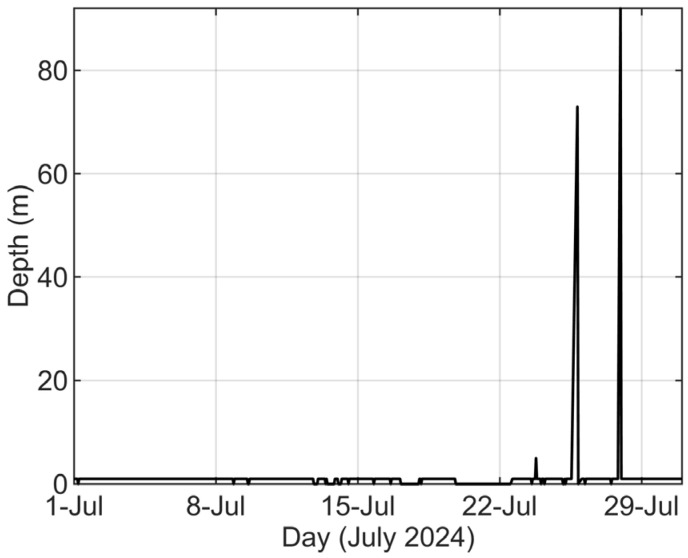
The Depth of the Communication Buoy.

The system commenced data transmission immediately after deployment. Over the initial 42-day observation period, the shore station achieved a data reception rate of 90.45%. As shown in Figure 5, the depth data from the communication buoy in July is analyzed. The buoy remained above sea level most of the time, but occasionally dipped into the water, which compromised its communication effectiveness. Analysis of buoy communication data reveals a significant correlation between the frequency of communication interruptions and the astronomical spring tide cycle, with failures primarily occurring around the periods of new moon and full moon spring tides. The fundamental cause of this phenomenon likely lies in the active internal solitary waves in the South China Sea. During the passage of internal solitary waves, extremely strong sudden currents are generated at the sea surface, which will pull the buoy into the sea. In the future, we will optimize the anchor system design and consider adopting longer mooring line or bigger buoyancy to enhance its stability under strong current impacts. Simultaneously, we will strengthen communication system redundancy to enable automatic data re-transmission upon recovery, fundamentally improving system reliability and data acquisition rates.

### 3.4. Analysis of Hydrological Data Products

The collected data successfully captured the detailed hydrological structure of the water column. The temperature-salinity profiles from the 19 sensors revealed a pronounced thermocline and the characteristic salinity minimum layer, consistent with historical observations in the region. The simultaneous measurements from the two ADCPs provided a continuous, high-resolution view of the current velocity profile, clearly showing the strengthening of the subsurface current maximum and its vertical shear during the study period.

#### 3.4.1. Temperature-Depth Profile

**Figure 6:**
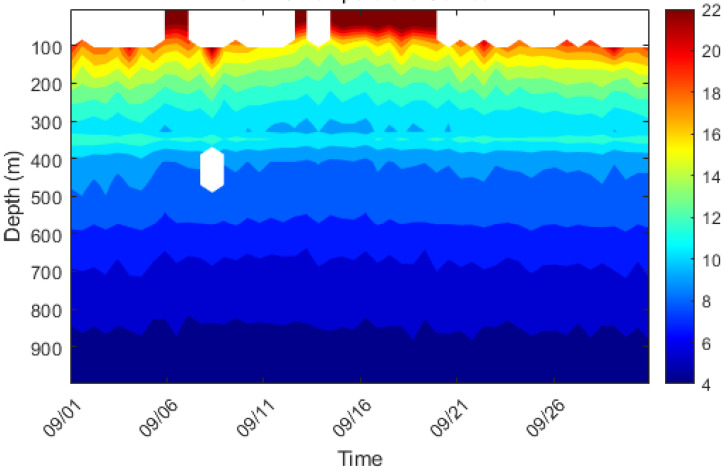
Typical Temperature-Depth Profile (the white section denotes data of poor quality).

As shown in Figure 6, the real-time moored buoy system constructed in this study successfully acquired high-resolution vertical temperature profiles in the northern South China Sea. The observational data clearly reveal the typical marine thermal stratification structure in this sea area. The upper mixed layer extends from the sea surface to a depth of approximately 50–60 m, where water temperature uniformly decreases from about 22 °C to 20 °C with a relatively small vertical gradient. This indicates that the water in this layer is thoroughly mixed under the influence of dynamic processes such as wind and wave mixing, forming relatively homogeneous surface water. The main thermocline extends from approximately 60 to 800 m depth, where temperature decreases sharply with depth—plunging abruptly from 20 °C to about 5 °C. This layer exhibits the most dramatic vertical temperature variation. Its strong density gradient forms a barrier to vertical exchange, exerting a decisive influence on the transport of energy and matter within the ocean.

#### 3.4.2. Salinity Profile

**Figure 7:**
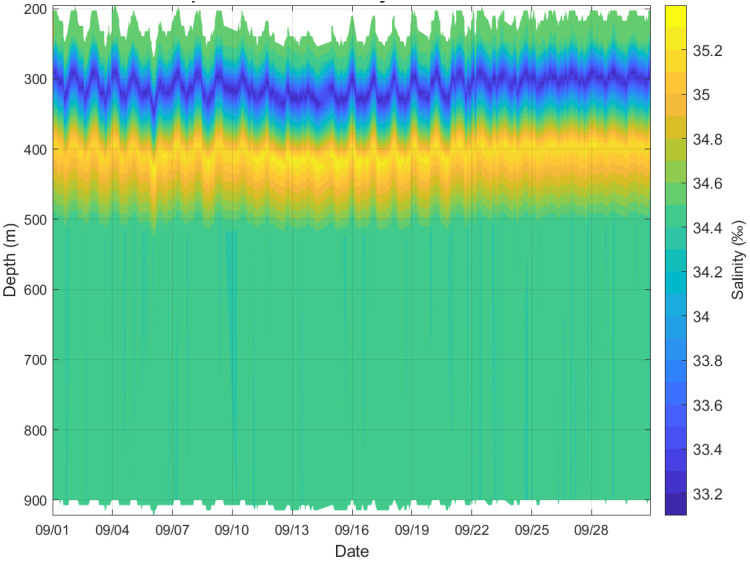
Typical Salinity-Depth Profile.

As shown in Figure 7, the system’s salinity observations reveal the complex vertical structure and temporal variability within the study area. The surface layer, characterized by low salinity water, exhibits relatively low salinity (approximately 33.6–34.2) at depths shallower than 50 m and demonstrates high temporal variability. This low-salinity feature likely originates from summer precipitation or freshwater input from the surrounding continental shelf. A pronounced hypersaline core layer exists between approximately 100 and 300 m depth, characterized by salinities exceeding 34.8. Maximum salinities within this core layer can surpass 35.0. This represents a signature feature of North Pacific Tropical Water (NPTW) intrusion into the South China Sea. This hypersaline mass enters through conduits such as the Luzon Strait, subsequently sinking and spreading throughout the South China Sea interior. Deep-water salinity distribution: Below the hypersaline core layer, salinity gradually decreases with depth. At depths greater than approximately 800 m, salinity stabilizes around 34.4–34.5, consistent with the characteristics of deep-water in the South China Sea.

#### 3.4.3. Temperature-Salinity Diagram

**Figure 8:**
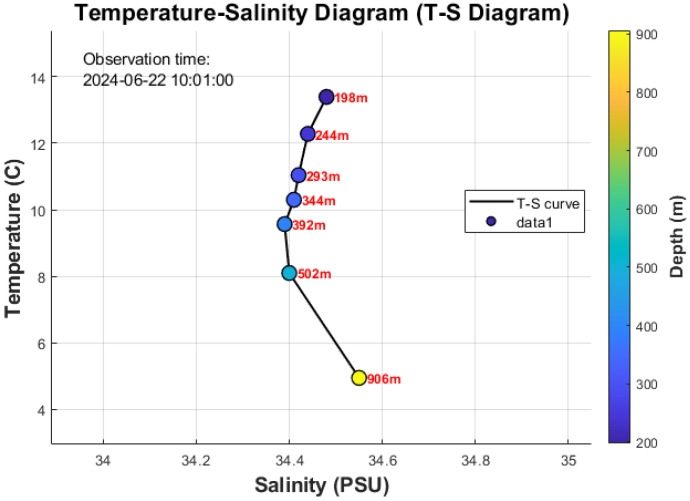
Temperature-Salinity (T-S) diagram.

The T-S diagram (Figure 8) provides a integrated view of the water mass structure in the northern South China Sea. The data points, colored by depth, form a tight, coherent curve that aligns well with the known T-S characteristics of this region [26]. The temperature and salinity distribution ranges of this water mass are 4.95–13.39 °C and 34.39–34.55‰, respectively. This water mass is the least altered oceanic-type water mass in the South China Sea region, retaining the fundamental characteristics of mid-depth water masses in the western Pacific. The stability and clarity of this T-S relationship, derived from our multi-sensor array, validates the high quality and internal consistency of the data delivered by the inductive telemetry system.

#### 3.4.4. Current Profile

**Figure 9:**
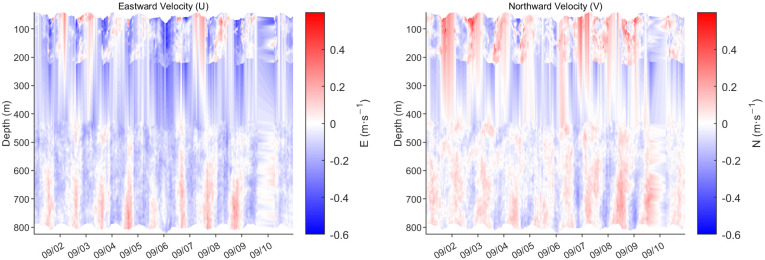
Current profile observed during the passage of Typhoon Yagi.

**Figure 10:**
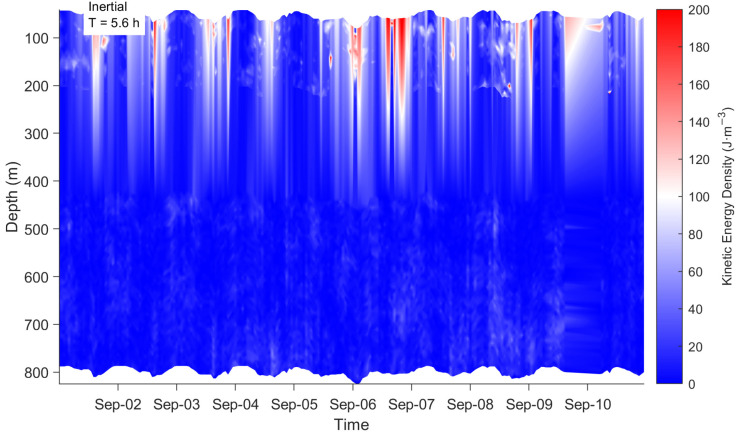
The kinetic energy during the passage of Typhoon Yagi.

Figure 9 presents high-resolution ocean current profile data acquired during the passage of Typhoon Yagi in 1–10 September 2024, analyzing the dynamic response of the upper ocean to the intense typhoon. Observations reveal a strong surface current pulse of up to 0.6 m/s in the northward component during the typhoon’s core passage, rapidly decaying with depth. Figure 10 shows the distribution of near-inertial energy during the typhoon. It can be observed that energy is primarily confined within the upper 200 m, exhibiting distinct near-inertial oscillatory characteristics. The signal period is approximately 5.6 h, nearly identical to the local inertial period, indicating that near-inertial motion dominates the flow field. This exhibits typical wind-driven flow structure and near-inertial oscillation characteristics. Concurrently, the eastward component velocity shows frequent directional shifts correlated with the typhoon’s rotating wind field. The overall flow field was dominated by a northward component, indicating the observation point was located on the typhoon’s right-side path—the region receiving the strongest energy input. This study visually reveals how Typhoon Yagi injected immense energy into the upper ocean and drove intense currents, providing a key case study for understanding typhoon-ocean interactions.

## 4. Discussion

The successful deployment and operation of the real-time mooring system in the South China Sea demonstrate the efficacy of the inductive telemetry + Tiantong satellite solution for deep-sea observation. The core achievement of this study lies in establishing a highly reliable, continuous, and near-real-time data link from the seabed to the shore station, a feat that has been a significant challenge for conventional approaches.

The performance of our system stands in stark contrast to existing real-time telemetry schemes. Firstly, compared to systems relying on acoustic modems, e.g., ref. [20], our inductive coupling method eliminates the inherent vulnerabilities of the underwater acoustic channel, such as signal attenuation, multi-path interference, and bio-fouling noise. This is evidenced by the sustained data reception rate of >90% over the deployment period. Acoustic systems, while capable of greater depths, often suffer from unpredictable data packet loss and require high-power consumption for transmission, compromising both data integrity and operational longevity. Our approach provides a stable, low-power, and high-fidelity connection akin to a “virtual cable,” ensuring the complete transmission of high-resolution datasets from multiple sensors.

Secondly, when compared to mobile profiling platforms (e.g., MMPs or SeaCyclers [11]), our fixed-depth, multi-sensor array provides a true synoptic view of the entire water column at a single moment. Profilers offer high vertical resolution but at the cost of poor temporal resolution for any given depth, potentially aliasing rapid dynamic processes like internal solitary waves or frontal movements. Furthermore, the mechanical complexity of profilers, with their moving parts and winching systems, presents a non-trivial risk of failure during long-term deployments. Our stationary design boasts greater mechanical simplicity and reliability, which is paramount for year-long unattended operations.

Lastly, against pop-up buoy systems, our solution offers genuine real-time capability. While pop-up buoys excel in covert, long-term data storage and are low-risk during their submerged phase, they provide only retrospective data delivery after a significant delay (weeks or months). This makes them unsuitable for time-critical applications such as ocean forecasting, typhoon monitoring, or rapid response to oceanic events. Our system’s <15 min latency is a transformative feature for operational oceanography and dynamic process studies.

The capability of our system to deliver full-water-column, real-time thermohaline and current profiles with high vertical resolution opens up new possibilities for studying submesoscale processes and validating high-resolution numerical models. For instance, the detailed structure of the thermocline and halocline captured by our 19 sensors provides a ground truth for model simulations of stratification. Furthermore, the ability to resolve near-inertial oscillations and their vertical propagation, as hinted at in the current response to Typhoon Yagi, is crucial for understanding the pathway of wind energy into the ocean interior and its role in driving mixing.

Nevertheless, the system’s surface expression remains a vulnerability, exposing it to potential damage from fishing activities, vessel traffic, and extreme sea states. To enhance resilience against strong internal waves and extreme surface conditions, we plan to increase buoyancy and extend mooring length in future deployments, which will help maintain antenna elevation and alignment. Furthermore, to mitigate data loss during communication interruptions, the system will be enhanced with robust data buffering and automatic retransmission capabilities, ensuring data integrity even during prolonged link outages. Future iterations could also explore hybrid communication strategies, such as combining inductive telemetry with a submerged, satellite-ready buoy that only surfaces briefly for data transmission, further mitigating surface-related risks.

## 5. Conclusions

This paper develops a deep-sea real-time communication moored buoy system, providing a reliable platform for long-term real-time observation in the deep ocean. Based on inductive telemetry technology (EUM6000 module) and Tiantong satellite communication, this deep-sea real-time transmission moored buoy system integrates 25 sensors including CTD and ADCP. It achieves continuous observation at a water depth of 1247 m for ≥1 year with a data reception rate >90%. Multi-sensor data fusion enhances profiling resolution for layered observations at 50–1000 m. This mooring system holds promising applications in ocean dynamics research, climate model validation, and disaster early warning. This system demonstrates a viable and robust pathway for real-time data transmission from the deep sea, serving not only as a standalone observatory but also as a potential prototype for future nodes in a comprehensive, integrated ocean observation network. Future studies may explore the impact of biofouling on buoyancy, long-term reliability testing of sealing components under deep-sea high-pressure conditions, and system safety.

## Figures and Tables

**Table 1:** Sensor inventory and key specifications deployed on the deep-sea mooring system.

Sensor Type	Model	Quantity	Key Specifications	Depth Rating
Inductive CTD	SBE37-SM	12	Accuracy: Temp.: ±0.002 °C; Cond.: ±0.003 mS/cm; Sal.: ±0.005 PSU	7000 m
Inductive T-D	OceanPlot TD-IM	8	Accuracy: Temp.: ±0.002 °C; Depth: ±0.05% FS	2000 m
Inductive Current Meter	ZPulseIM	3	Accuracy: ±1% ± 0.5 cm/s; Range: 0–5 m/s	6000 m
Acoustic Doppler Current Profiler (ADCP)	Teledyne Workhorse Long Ranger 75 kHz	2	Max Range: 600 m; Accuracy: ±1% ± 0.5 cm/s	1500 m

**Table 2:** Key specifications of the EUM6000 inductive telemetry module.

Parameter	Specification	Note/Implication
Operating Depth	6000 m	Compatible with abyssal seafloor deployments.
Data Transmission Rate	4800 bps	Adequate for high-frequency data from CTD, ADCP, and pressure sensors.
Maximum Range	6000 m	Supports data transmission over the full length of deep-sea moorings.
Power Consumption	<1 W (active)	Essential for long-term (≥1 year) battery-powered operation.
Communication Interface	RS-232/Inductive Coupling	Enables seamless connection to both standard sensors and the inductive cable.
Number of Slave Units Supported	Up to 32	Allows for dense multi-sensor integration on a single mooring line.

## Data Availability

Data is available upon reasonable request to the corresponding author.

## Abbreviations

The following abbreviations are used in this manuscript:

ADCP	Acoustic Doppler Current Profiler
TD	Temperature–Depth sensor
TD-IM	Temperature–Depth sensor with an Inductive Modem
CTD	Conductivity–Temperature–Depth sensor
CTD-IM	Conductivity–Temperature–Depth sensor with an Inductive Modem
